# School-Based Prevention of Screen-Related Risk Behaviors during the Long-Term Distant Schooling Caused by COVID-19 Outbreak

**DOI:** 10.3390/ijerph18168561

**Published:** 2021-08-13

**Authors:** Kateřina Lukavská, Václav Burda, Jiří Lukavský, Michaela Slussareff, Roman Gabrhelík

**Affiliations:** 1 Department of Addictology, First Faculty of Medicine, Charles University, 12000 Prague, Czech Republic; katerina.lukavska@lf1.cuni.cz (K.L.); burdavac@fel.cvut.cz (V.B.); Michaela.Slussareff@ff.cuni.cz (M.S.); 2 Department of Addictology, General University Hospital in Prague, 12000 Prague, Czech Republic; 3 Department of Psychology, Faculty of Education, Charles University, 11000 Prague, Czech Republic; 4 Department of Cybernetics, Czech Technical University in Prague, 16627 Prague, Czech Republic; 5 Department of Psychology, Faculty of Arts, Charles University, 11638 Prague, Czech Republic; lukavsky@praha.psu.cas.cz; 6 Institute of Information Studies and Librarianship, Faculty of Arts, Charles University, 11638 Prague, Czech Republic

**Keywords:** school-based prevention, adolescents, children, cross-sectional survey, excessive/problematic use of screens, internet, distant schooling

## Abstract

The COVID-19 outbreak and related restrictions meant a higher incidence of screen-related risk behaviors in both children and adolescents. Our goal was to assess the perceived importance and extent of school-based preventions related to these risks during the long-term, nation-wide distant schooling period in the Czech Republic. The online survey was responded to by the school-based prevention specialists (N = 1698). For the analysis, within-subject analysis of variance (ANOVA) and binominal logistic regression were used. At-risk internet use and cyber-bullying were perceived as pressing, but other risks, for example, excessive internet use or the use of cyberpornography, received substantially less priority. The differences in all grades were significant and moderate to large (η^2^G between 0.156 and 0.288). The proportion of schools which conducted prevention interventions of screen-related risks was low (between 0.7% and 27.8%, depending on the grade and the type of the risk). The probability of delivering prevention intervention was in all grades significantly predicted by the presence of screen-related problems in pupils (OR 3.76–4.88) and the perceived importance of the screen-related risks (OR 1.55–1.97). The limited capacity of schools to deliver prevention interventions during distant schooling as well as the low awareness and impaired ability to recognize the importance of some screen-related risks should be addressed.

## 1. Introduction

The COVID-19 pandemic has significantly affected almost all areas of life, [1] including education. In this unprecedented situation, many countries worldwide have been forced to close schools, and later to switch from present to distant schooling in all school grades. During the spring 2020 outbreak, it has been estimated that more than 1 billion children worldwide were out of school or childcare [2].

Several studies have demonstrated that during the COVID-19 period the use of “screens” by children and adolescents has increased. Studies reported increased time spent on smartphones [3] and on social media [3,4], and more frequent incidences of gaming disorder symptoms [5] compared with the pre-COVID-19 period. Prevalence studies conducted during the COVID-19 period have also emphasized the high prevalence of problematic internet use among youths, and the necessity of addressing this issue through preventative and educational efforts [6,7,8].

Both problematic (addiction-like) use of screens and at-risk use of screens, such as cyber-bullying and pornography use, have been reported to have negative impacts on users [9,10,11,12,13,14]. These screen related problems were present in children even before the COVID-19 pandemic [15,16,17,18,19,20]. Therefore, the prevention of screen addictions and online risks is increasingly important. The key question is: who should provide such prevention? In theory, screen activities in children might be regulated by their parents [21], but the currently available studies suggest that the effects of parenting within the area of screen-use are limited [22,23,24,25,26]. In standard schooling conditions, the prevention of risk behaviors in children is one of the important functions of schools. In the Czech Republic, it is compulsory for each primary, secondary, and high school to designate a specialist who manages (planning, intervening, and evaluating) activities related to the prevention of risk behaviors; these are most often teachers who have received specialized extensive training in prevention [27].

School-based prevention interventions are shown to effectively address various types of risk behaviors [28,29,30,31,32] and various subgroups [33,34], but prevention programs targeting online risks remain scarce [35]. To sum up, the screen-related risks seem to be accentuated by the situation of distant schooling; at the same time, schools may have a limited ability to recognize and address (screen-related) risks in children and adolescents. Studies examining how the pandemic lockdown has affected the schools’ motivation and capacity to conduct prevention interventions are missing.

To address this gap, a rapid survey assessing the school-based prevention during the nation-wide school lockdown caused by the fall wave of the COVID-19 outbreak was conducted. This study utilized data concerning screen-related risk behaviors (i.e., cyber-bullying, at-risk internet use, online gambling, excessive/problematic use of cyber-pornography, and excessive/problematic use of internet and games). Specifically, we aimed to estimate (1) the relative importance of addressing these risks in school-based preventions according to the school-based prevention specialists, (2) the prevalence of screen-related problems in pupils and students, (3) the extent to which distant prevention interventions focused on screen-related risks have been provided.

## 2. Materials and Methods

### 2.1. Design

Nation-wide rapid survey method.

### 2.2. Participants

In total, 1778 school-based prevention specialists (SPS) participated in our survey. Eighty of them have been excluded due to a large number of missing values (more than 35% of all values in the questionnaire). The final sample consisted of 1698 SPS who provided a total of 2453 unique questionnaires. Specifically, we collected data concerning primary school grades (1–5) (n = 1068), secondary school grades (6–9) (n = 934) and high school grades (n = 451). The difference in the number of participating SPS and the number of questionnaires reflects that some SPS act as prevention specialists in both primary and secondary school grades. Thus, 926 SPS filled out only one questionnaire; 664 SPS filled the questionnaire for both primary and secondary school grades (1–5 and 6–9); and 87 SPS for secondary (6–9 grades) and high school grades. In the Czech Republic, only a minority of schools combine all three school levels; 21 SPS filled out the questionnaire for primary, secondary, and high schools.

### 2.3. Procedure

The Czech Republic closed all schools in reaction to the COVID-19 outbreak twice in 2020. First, in spring 2020, all schools were closed as of 11 March, 2020 and the restrictions began to be slowly lifted by 20 April, 2020, and again on 14 October 2020 when restrictions continued until the end of November. Data were collected during the second lockdown, between 16 and 27 November 2020.

The questionnaire was developed specifically for this study and suited for online administration. The time needed to complete the questionnaire was approximately 15 min. The questionnaire consisted of four parts, each assessing different aspects of school-based prevention in the situation of long-term distant schooling: (i) quality of communication between teachers, students, and their parents; (ii) perceived importance of addressing risk behaviors in school-based prevention interventions; (iii) prevalence of risk behaviors (problems) in students, and the extent of delivering prevention interventions; (iv) barriers related to delivering distant school-based prevention interventions.

The questionnaire was administered via the online platform System of Records of Preventive Activities (SRPA). SRPA, launched in the school year 2014/2015, is a nation-wide online platform which provides the technical infrastructure needed to enter and record information about the implemented prevention interventions which target all main types of risk behaviors in schools in the Czech Republic. Via SRPA, we could quickly access the SPS. The main reason for using SRPA was that it directly targets our specific sample of school-based prevention specialists.

SPS from all primary, secondary, and high schools in the country were asked directly to participate via email twice, on 16 November 2020 and then in a follow-up email on 24 November, 2020. The email, prepared by the authors of the study, was sent out by all 14 regional prevention coordinators (there are 14 regions in Czechia). Another electronic letter was sent to the management of all schools in the country. The study was supported by the Governmental Office of the Czech Republic, the key national-level ministries, and the Czech Professional Society for Prevention.

After logging into SRPA, the SPS followed the instructions; after selecting the ‘show questionnaire’ button, the responsible SPS completed and submitted the questionnaire on a voluntary basis. All participants provided permission of their participation.

### 2.4. Instruments

This study utilized data from the second and third part of the online questionnaire (see above), namely:

- the perceived importance in providing prevention interventions during distant schooling in general (item “How important do you consider conducting prevention interventions during the distance schooling?”. Participants responded on a Likert scale from 1 = not at all to 5 = very much),
- the perceived importance in providing distant prevention interventions targeting screen-related risks (item “How important do you consider the prevention of [specific type of risk behavior] during distance schooling?”. Participants responded on a Likert scale from 1 = not at all to 5 = very much),
- the observed presence of risk behaviors (problems) in pupils and students during the distant schooling period (item “Try to estimate the total number of reported cases of [specific type of risk behavior] in students at your school during this wave of distance schooling”), and
- those prevention interventions conducted during the distant schooling (item “Have you conducted preventions of [specific type of risk behavior] during this wave of distance schooling?”. Participants responded yes/no).

The examined specific types of risk behaviors in this study were (a) cyber-bullying (cases of repeated use of electronic devices to maliciously cause harm to another person), (b) at-risk internet use (sharing sensitive data online, online communication with strangers, exposure to traumatic online content), (c) online gambling, (d) excessive/problematic use of (online) pornography, (e) excessive/problematic use of internet/games.

### 2.5. Data Analysis

First, the general perceived importance of providing school-based preventions in the situation of long-term distant schooling was established. Second, the perceived importance to address individual screen-related risks was assessed and differences between the risks were analyzed using the within-subject analysis of variance (ANOVA) and Bonferroni post hoc tests. To establish the effect sizes for ANOVA, both generalized eta-square (η^2^G) and partial eta-square (η^2^p) were computed according to recommended standards [36].

Third, using the subsample of 664 SPS that provided data both for grades 1–5 and 6–9, the perceived importance of addressing individual screen-related risks between grades (1–5 versus 6–9) were compared using the paired *t*-test and Cohen’s d for measuring effect sizes.

Fourth, the presence of screen-related problems in students during the relevant period was established.

Fifth, the percentage of schools that conducted any type of prevention interventions focused on any type of risk/problematic behavior was calculated. Then, the percentage of schools that delivered prevention interventions that focused on screen-related risk behaviors was ascertained and the differences between risk behaviors in the incidence of prevention interventions was established using within-subject ANOVA with Bonferroni post hoc tests.

Finally, using binominal logistic regression, we analyzed whether the perceived importance together with the presence of screen-related problems influenced the probability of conducting prevention interventions aimed at screen-related risks.

### 2.6. Missing Values

In the final data, the number of missing values was low: 2.67% in primary schools (grades 1–5), 2.76% in secondary schools (grades 6–9), and 2.22% in high school grades. The missing data were imputed with random values from the empirical distribution of the corresponding variable.

## 3. Results

### 3.1. The Perceived Importance of Delivering Prevention Interventions in the Situation of Distant Schooling

The perceived importance of conducting preventions during distant schooling generally was not very high. The mean values were 3.16 (SD = 1.12) in primary schools, 3.40 (SD = 1.11) in secondary schools, and 3.25 (SD = 1.10) in high school grades (response scale was from 1 to 5 with value “3” meaning “neither important nor unimportant”).

As for the individual screen-related behaviors, significant differences in their perceived importance were found (Table 1). To sum up, SPS perceived it as rather/very important to conduct preventions focused on at-risk internet use, cyber-bullying, and excessive use of internet/games, and significantly less important to conduct prevention interventions focused on online gambling and the use of pornography. In primary schools, the omnibus differences were significant with moderate effect: F(4,4268) = 936, *p* < 0.001, η^2^G = 0.288, η^2^p = 0.467; and all post hoc tests were significant (*p* < 0.001), except for the difference between cyber-bullying and internet/games use (*p* = 0.058). Similar results were also found for secondary schools, where the omnibus differences between behaviors were significant and the effect was moderate: F(4,3732) = 531, *p* < 0.001, η^2^G = 0.219, η^2^p = 0.363. All post hoc tests were significant (*p* < 0.001), except two: (1) cyber-bullying and internet/games use (*p* = 1.000), which were both perceived as rather/very important, and (2) gambling and pornography use (*p* = 0.130), which were both perceived as rather important/neither unimportant nor important. Additionally, for high-school grades, omnibus differences were significant and moderate: F(4,1800) = 189, *p* < 0.001, η^2^G = 0.156, η^2^p = 0.295; with all post hoc tests significant except for the difference between cyber-bullying and internet/games use (*p* = 1.000).

The perceived importance of delivering prevention interventions focused on screen-related risks was significantly higher for secondary school grades (6–9) compared to primary school grades (1–5) (Table 1). The differences were tested on the subsample of 664 SPS who filled out questionnaires for both the primary and secondary school grades. The differences were significant for all risk behaviors and the largest effects were observed for pornography use and online gambling (Table 2).

### 3.2. The Presence of Problematic Screen-Related Behaviors in Students during Distant Schooling

Only a minority of SPS reported some cases of risk behavior (any type) or mental health problems during the period followed: 86 SPS out of 1068 (8.1%) in primary school grades, 186 SPS out of 934 (19.9%) in secondary school grades, and 143 SPS out of 451 (31.7%) of high school grades.

As for screen-related risk behaviors, only 36 SPS out of 1068 (3.4%) in primary school grades, 82 SPS out of 934 (8.8%) in secondary school grades, and 42 high school SPS out of 451 (9.3%) reported some cases.

### 3.3. The Delivering of Preventive Interventions during Distant Schooling

The percentage of schools that conducted any prevention interventions (any form of intervention focused on any type of risk behavior or mental health) during the period that followed was quite low. In total, 445 schools (41.7%) conducted interventions in primary schools; 455 (48.7%) schools in secondary schools, and 221 schools (49.0%) in high school grades.

The frequency of conducting prevention interventions aimed at specific types of screen-related behaviors is shown in Table 3. The results were similar for all three grades (primary school, secondary school, high school). The relatively most frequent were prevention interventions focused on cyber-bullying (conducted in 22–28% of schools), followed by interventions on at-risk internet use. The least frequent were interventions focused on pornography use and gambling. The omnibus differences between behaviors were significant in all grades, but with small effects: in primary schools, F(4,4268) = 159, *p* < 0.001, η^2^G = 0.077, η^2^p = 0.130; in secondary schools, F(4,3732) = 168, *p* < 0.001, η^2^G = 0.084, η^2^p = 0.152; in high school grades, F(4,1800) = 64.5, *p* < 0.001, η^2^G = 0.072, η^2^p = 0.125. Most post hoc tests were also significant (Table 3).

The percentage of schools that conducted any prevention interventions aimed at screen-related risks was low. Which factors could increase the probability of conducting interventions was analyzed. Two predictors were assessed: the SPS perception of importance in conducting such interventions, and the presence of problematic screen-related behaviors. Both these factors were found to be significant predictors (Table 4). Results suggest that the chances of conducting prevention interventions was approximately four times higher if any screen-related problems were detected by the SPS. In comparison, the perceived importance of screen-related risks had a lower predictive power (OR between 1.6 and 2.0).

## 4. Discussion

Although screen-related risk behaviors were generally perceived as rather important to address by the school-based prevention specialists (SPS), there were significant differences between the various screen-related risks. At-risk internet use and cyber-bullying were reported as being of the highest importance, followed by the excessive/problematic use of internet/games. On the other hand, online gambling and the use of cyberpornography was perceived as the least important in all grades. SPS perceived the delivery of prevention interventions as more important for students attending secondary schools, i.e., children aged 11–16 years, compared to children attending primary school (aged 6–11 years). The proportion of schools that conducted prevention interventions focused on screen-related risks during long-term distant schooling caused by the fall 2020 wave of COVID-19 in the Czech Republic was low. It ranged between 0.7% to 27.8%, depending on the grade/age group and the type of screen-related risk behavior. The most frequently addressed risk behavior was cyber-bullying and at-risk internet use, while significantly fewer schools addressed the excessive use of the internet/games, and almost no schools delivered prevention interventions aimed at online gambling and pornography use. The probability of delivering prevention interventions focused on screen-related risks was affected by the recognized presence of screen-related problems in pupils and by the perceived importance of screen-related risks according to SPS. The reported presence of screen-related problems in pupils and students was low in all grades (only 3.4–9.3% of schools reported some cases).

The COVID-19 outbreak and the unprecedented restrictions adopted in many countries exposed some trends in the area of adolescent risk behavior that has been present for at least the last decade, e.g., the increasing incidence of problematic internet use and/or screen devices in children and adolescents. Significant differences between various screen-related risk behaviors were found in (i) the perceived importance of addressing them via school-based prevention interventions and (ii) in the number of delivered interventions during the period of long-term distant schooling. There seem to be several reasons for these differences. First, some of screen-related risk behaviors might be easier to detect than others in the school setting. Typically, the cyber-bullying and at-risk internet use represent problems with consequences that are visible within weeks or months. In addition, these risks have recently received much attention due to media coverage in the Czech Republic (e.g., popular documents or fictions such as the recent Emmy Award winner series #martyisdead). Conversely, some problems related to screen use may be subtle or may affect young people in the long-term, i.e., after leaving the (secondary or high) school. There are several examples, the first of which is the problematic (addictive) use of screens (e.g., internet, games, smartphones). Although the scientific debate on the nosology of these (and other) behavioral addictions is still in progress, there is no doubt that the use of screens is, in vulnerable populations, out of control and may cause functional impairment [9,14,17]. Problems with control over screen use may go unseen for a long time because parental control can to some extent substitute the child’s impaired self-regulation mechanisms. The second example is the use of online pornography, which, in our study, was the most underestimated and the least addressed risk. There is an ongoing debate as to the potential negative effects (e.g., sexual functioning problems) of pornography use in adults [10,18]. Although current studies suggested that a problematic use of pornography (PPU) develops in “only” up to 6% of pornography users, the lower age of users has been identified as a risk factor for PPU [11,12,19,20]. Moreover, it has been shown that the use of pornography influences both sexual attitudes and practices of adolescents [13,21]. All these effects of pornography—PPU and harmful attitudes toward sex, body, and relationships—emerge in the long term. Therefore, teachers and school-based prevention specialists or school psychologists may easily fail to recognize the importance of addressing the use of (online) pornography, unless they are systematically sensitized to this type of risk behavior.

The reported presence of any type of screen-related problem was very low. In primary schools, only 3.4% of schools reported some cases; in older students, the problems were observed by 8.8%, respectively, 9.3% of SPS. Such low numbers did not correspond with the prevalence of screen-related pathological behaviors (e.g., problematic internet use, internet gaming disorder, cyber-bullying) in children and adolescents as estimated by studies conducted during the COVID-19 pandemic [2,3,4,5,6,7,8] or before [15,16,17,18,19,20,22,23,24,25,26,27]. This suggests that the ability of schools to detect problems is limited, at least in the situation of distant schooling. Our study demonstrated that the detection of screen-related problems in schools is the important predictor of delivering school-based prevention interventions focused on these risks. Therefore, the limited ability of schools to detect (screen-related) problems may negatively influence the extent to which they conduct preventions. Therefore, special attention should be given to the development, evaluation, and implementation of screening instruments, which will be easy to use in school settings. However, the sole existence of such instruments does not guarantee their appropriate use and SPS should be trained in their use in regular school prevention practice.

Significant differences between primary (1–5) and secondary (6–9) grades in the perceived importance of addressing screen-related risks were found. The difference was most pronounced in the use of pornography, which was not perceived as an important topic at primary level (children aged 6–11 years). However, the initial age of encountering pornography content is low. Based on the most recent EU Kids Online report [37], in the EU, 15% of 9–11 year-old internet users have seen inappropriate sexual images (in the Czech Republic 21%), while 68% of them found the content upsetting. Thus, addressing the online pornography as late as in secondary grades (children older than 11 years) is not optimal. Similarly, the addictive use of screens needs to be prevented by timely interventions focused on healthy screen use habits and the strengthening of self-regulation processes [9,38]. During the period of distant schooling, only 14–17% of schools conducted any prevention focused on excessive use of the internet/games, which does not seem to be a sufficient response to the COVID-19-related increase in the prevalence of the problematic use of screens in children and adolescents.

### Strengths and Limitations

By using and accessing the national registry (SRPA) on school-based prevention activities, it was possible to establish a national cohort represented by a large sample (almost 32% of all 5373 schools in Czechia). We were able to assess the current situation during the time of applied distant schooling and nationwide lockdown on a large nationwide sample in a very short time—exclusively during November 2020. As the data were collected during only a two-week period, the homogeneity of the responding schools’ conditions was high, even in the dynamic situation of the pandemic. Last but not least, the study provides insights into the screen-related risk behaviors for all three stages of the school system, children aged 6–19 years.

One limitation worth noting is that SRPA represented a higher threshold for those schools and SPS that were not registered in SRPA before the study. Moreover, the data collection period was short (two weeks). These two factors may have resulted in the involvement of schools that were more motivated in the area of the prevention of risk behaviors. Notwithstanding this sampling bias, the involvement in the prevention of screen risks in the participating schools was alarmingly low. We could hypothesize that the situation in the non-participating, and probably less motivated, schools was even worse. In addition, the study did not assess the quality of the prevention interventions. The limited number of interventions that were reported by participating schools might not be effective enough to address the extensity and intensity of the screen-related risks. Additionally, the observed associations between the presence of problems, importance ratings and delivered interventions may reflect the reverse causal link: SPS start to perceive some activities as important only after a case requiring intervention appears. Finally, data on the prevalence of screen-related problems must be interpreted with caution since these estimates are expert-based and not reported by the children themselves.

## 5. Conclusions

School-based prevention specialists recognize the importance of addressing most of the examined screen-related risk behaviors, with the exception of online gambling and pornography. It is necessary to bring these topics to the attention of these professionals.

A profound discrepancy was found between the perceived importance of delivering prevention interventions focused on various screen-related risks and the number of interventions delivered during distant schooling. School-based prevention specialists recognized the necessity of preventively addressing most screen-related risks, but few of them delivered any prevention interventions focused on these risks. The important impulse for conducting prevention is the revealed presence of screen-related problems in students, but the ability of schools to detect these problems seems to be limited. School-based prevention specialists (i) should be provided with quality and easy-to-use screening instruments for various types of screen-related risk behaviors and various age groups and (ii) should be instructed to systematically screen for these risk behaviors.

The current pandemic situation has increased the risk of screen-related problems in children and adolescents, which is not countered by an increased prevention activity of schools. Therefore, the fast development of school-based prevention policies and prevention programs focused on various screen-related risk behaviors should be the high priority of policymakers, scholars, and professionals in the field of prevention.

## Figures and Tables

**Table 1:** The perceived importance of conducting prevention interventions focused on screen-related risks during the fall 2020 wave of long-term distant schooling caused by COVID-19 in primary (n = 1068), secondary (n = 934), and high schools (n = 451). The response scale was 1 to 5.

	Primary School (Grades 1–5)		Secondary School (Grades 6–9)		High School	
	Means	Post Hoc Tests	Means	Post Hoc Tests	Means	Post Hoc Tests
	**(SD)**		**(SD)**		**(SD)**	
1. Cyber-bullying	4.36	>3,4; =5; <2	4.56	>3,4; =5; <2,	4.56	>3,4; =5; <2
	(0.904)		(0.737)		(0.882)	
2. At-risk internet use	4.52	>1,3,4,5	4.73	>1,3,4,5	4.59	>1,3,4,5
	(0.822)		(0.589)		(0.688)	
3. Online gambling	3.08	>4; <1,2,5	3.70	=4; <1,2,5	3.78	>4; <1,2,5
	(1.33)		(1.14)		(1.11)	
4. Pornography use	2.87	<1,2,3,5	3.62	=3; <1,2,5	3.49	<1,2,3,5
	(1.39)		(1.13)		(1.12)	
5. Internet/games use	4.26	>3,4; =1; <2	4.54	>3,4; =1; <2	4.35	>3,4; =1; <2
	(0.902)		(0.711)		(0.845)	

**Table 2:** Within-subject between-grade (primary versus secondary school) differences in the perceived importance of conducting prevention interventions focused on screen-related risks during the fall 2020 wave of long-term distant schooling caused by COVID-19 (n = 664).

	t (df = 663)		Mean Difference	SE Difference	Cohen d
Cyber-bullying	9.58	***	0.208	0.0217	0.372
At-risk internet use	10.21	***	0.229	0.0224	0.396
Online gambling	19.34	***	0.745	0.0385	0.751
Pornoraphy use	21.66	***	0.895	0.0413	0.841
Internet/games use	13.01	***	0.330	0.0253	0.505

*** *p* < 0.001. This analysis was conducted on the sample of school-based prevention specialists (SPS) who provided data concerning both primary and secondary levels of the same elementary school. Pairwise *t*-tests were used.

**Table 3:** Percentage of schools that conducted prevention interventions of screen-related risk behaviors during the fall 2020 wave of COVID-19-related distant schooling period in primary (n = 1068), secondary (n = 934), and high schools (n = 451). Response scale was 1 to 5.

	Primary School (Grades 1–5)		Secondary School (Grades 6–9)		High School	
	% of School	Post Hoc Tests	% of School	Post Hoc Tests	% of School	Post Hoc Tests
1. Cyber-bullying	22.8	>2,3,4,5	27.8	>2,3,4,5	21.7	>3,4,5; =2
2. At-risk internet use	19.1	>3,4,5; <1	23.4	>3,4,5; <1	20.8	>3,4,5; =1
3. Online gambling	2.0	=4; <1,2,5	3.6	=4; <1,2,5	2.7	=4, <1,2,5
4. Pornography use	0.7	=3; <1,2,5	2.2	=3; <1,2,5	1.1	=3, <1,2,5
5. Internet/games use	13.7	>3,4; <1,2	16.8	>3,4; <1,2	15.5	>3,4; <1,2

**Table 4:** Results of binominal logistic regression model predicting the conducting of prevention intervention(s) aimed at screen-related risk behaviors in primary (n = 1068), secondary (n = 934), and high schools (n = 451).

Predictors	Estimate		SE	z	OR	95% CI	
						Lower	Upper
*Primary school (Grades 1–5)*							
Intercept	−2.59	***	0.36	−7.14	0.07	0.04	0.15
Perceived importance of screen-related risks	0.44	***	0.09	4.86	1.55	1.30	1.86
Presence of screen-related problems							
Yes	1.56	***	0.36	4.30	4.78	2.34	9.74
*Secondary school (Grades 6–9)*							
Intercept	−3.36	***	0.55	−6.13	0.03	0.01	0.10
Perceived importance of screen-related risks	0.61	***	0.13	4.85	1.84	1.44	2.36
Presence of screen-related problems							
Yes	1.49	***	0.25	5.90	4.42	2.70	7.25
*High school*							
Intercept	−3.76	***	0.75	−5.04	0.02	0.01	0.10
Perceived importance of screen-related risks	0.68	***	0.17	3.87	1.97	1.40	2.77
Presence of screen-related problems							
Yes	1.35	***	0.34	3.93	3.86	1.97	7.57

*** *p* < 0.001.

## Data Availability

The data presented in this study are openly available in OSF at https://osf.io/ryz9v/, DOI 10.17605/OSF.IO/58TQY (accessed on 10 August 2021).

## References

[1] Hermassi S., Sellami M., Salman A., Al-Mohannadi A., Bouhafs E.G., Hayes L.D., Schwesig R. (2021). Effects of COVID-19 Lockdown on Physical Activity, Sedentary Behavior, and Satisfaction with Life in Qatar: A Preliminary Study. IJERPH.

[2] Cluver L., Lachman J.M., Sherr L., Wessels I., Krug E. (2020). Parenting in a time of COVID-19. Lancet.

[3] Chen I.-H., Chen C.-Y., Pakpour A.H., Griffiths M.D., Lin C.-Y., Li X.-D., Tsang H.W.B. (2021). Problematic internet-related behaviors mediate the associations between levels of internet engagement and distress among schoolchildren during COVID-19 lockdown: A longitudinal structural equation modeling study. J. Behav. Addict..

[4] Fernandes B., Biswas U.N., Tan-Mansukhani R., Vallejo A., Essau C.A. (2020). The impact of COVID-19 lockdown on internet use and escapism in adolescents. Rev. Psicol. Clínica Con. Niños Y Adolesc..

[5] Teng Z., Pontes H.M., Nie Q., Griffiths M.D., Guo C. (2021). Depression and anxiety symptoms associated with internet gaming disorder before and during the COVID-19 pandemic: A longitudinal study. J. Behav. Addict..

[6] Dong H., Yang F., Lu X., Hao W. (2020). Internet Addiction and Related Psychological Factors Among Children and Adolescents in China During the Coronavirus Disease 2019 (COVID-19) Epidemic. Front. Psychiatry.

[7] Gómez-Galán J., Martínez-López J., Lázaro-Pérez C., Sarasola Sánchez-Serrano J.L. (2020). Social Networks Consumption and Addiction in College Students during the COVID-19 Pandemic: Educational Approach to Responsible Use. Sustainability.

[8] Lin M.-P. (2020). Prevalence of Internet Addiction during the COVID-19 Outbreak and Its Risk Factors among Junior High School Students in Taiwan. IJERPH.

[9] Perales J.C., King D.L., Navas J.F., Schimmenti A., Sescousse G., Starcevic V., van Holst R.J., Billieux J. (2020). Learning to lose control: A process-based account of behavioral addiction. Neurosci. Biobehav. Rev..

[10] Bőthe B., Tóth-Király I., Griffiths M.D., Potenza M.N., Orosz G., Demetrovics Z. (2021). Are sexual functioning problems associated with frequent pornography use and/or problematic pornography use? Results from a large community survey including males and females. Addict. Behav..

[11] Bőthe B., Lonza A., Štulhofer A., Demetrovics Z. (2020). Symptoms of Problematic Pornography Use in a Sample of Treatment Considering and Treatment Non-Considering Men: A Network Approach. J. Sex. Med..

[12] Bőthe B., Tóth-Király I., Potenza M.N., Orosz G., Demetrovics Z. (2020). High-Frequency Pornography Use May Not Always Be Problematic. J. Sex. Med..

[13] Raine G., Khouja C., Scott R., Wright K., Sowden A. (2020). Pornography use and sexting amongst children and young people: A systematic overview of reviews. Syst. Rev..

[14] Fineberg N., Demetrovics Z., Stein D., Ioannidis K., Potenza M.N., Grünblatt E., Brand M., Billieux J., Carmi L., King D.I. (2018). Manifesto for a European research network into Problematic Usage of the Internet. Eur. Neuropsychopharmacol..

[15] Bányai F., Zsila Á., Király O., Maraz A., Elekes Z., Griffiths M.D., Andreassen C.S., Demetrovics Z. (2017). Problematic Social Media Use: Results from a Large-Scale Nationally Representative Adolescent Sample. PLoS ONE.

[16] Cheng C., Li A.Y. (2014). Internet Addiction Prevalence and Quality of (Real) Life: A Meta-Analysis of 31 Nations Across Seven World Regions. Cyberpsychol. Behav. Soc. Netw..

[17] Demetrovics Z., Király O., Koronczai B., Griffiths M.D., Nagygyörgy K., Elekes Z., Tamás D., Kun B., Kökönyei G., Urbán R. (2016). Psychometric Properties of the Problematic Internet Use Questionnaire Short-Form (PIUQ-SF-6) in a Nationally Representative Sample of Adolescents. PLoS ONE.

[18] Kuss D.J., Griffiths M.D., Karila L., Billieux J. (2014). Internet addiction: A systematic review of epidemiological research for the last decade. Curr. Pharm. Des..

[19] Lukavská K., Vacek J., Gabrhelík R. The effects of parental control and warmth on problematic internet use in adolescents: A prospective cohort study. JBA.

[20] Pápay O., Urbán R., Griffiths M.D., Nagygyörgy K., Farkas J., Kökönyei G., Felvinczi K., Oláh A., Elekes Z., Demetrovics Z. (2013). Psychometric Properties of the Problematic Online Gaming Questionnaire Short-Form and Prevalence of Problematic Online Gaming in a National Sample of Adolescents. Cyberpsychol. Behav. Soc. Netw..

[21] Petruzelka B., Vacek J., Gavurova B., Kubak M., Gabrhelik R., Rogalewicz V., Bartak M. (2020). Interaction of Socioeconomic Status with Risky Internet Use, Gambling and Substance Use in Adolescents from a Structurally Disadvantaged Region in Central Europe. IJERPH.

[22] Becoña E., Martínez Ú., Calafat A. (2013). Parental permissiveness, control, and affect and drug use among adolescents. Psicothema.

[23] Collier K.M., Coyne S.M., Rasmussen E.E., Hawkins A.J., Padilla-Walker L.M., Erickson S.E., Memmott-Elison M.K. (2016). Does parental mediation of media influence child outcomes? A meta-analysis on media time, aggression, substance use, and sexual behavior. Dev. Psychol..

[24] Elsaesser C., Russell B., Ohannessian C.M., Patton D. (2017). Parenting in a digital age: A review of parents’ role in preventing adolescent cyberbullying. Aggress. Violent Behav..

[25] Nielsen P., Favez N., Liddle H., Rigter H. (2019). Linking parental mediation practices to adolescents’ problematic online screen use: A systematic literature review. J. Behav. Addict..

[26] Nielsen P., Favez N., Rigter H. (2020). Parental and Family Factors Associated with Problematic Gaming and Problematic Internet Use in Adolescents: A Systematic Literature Review. Curr. Addict. Rep..

[27] Miovsky M., Skacelova L., Cablova L., Vesela M., Zapletalova J. (2012). School-based prevention of risk behaviour: Proposed structure, scope, and content of the basic preventive programme [Návrh struktury, rozsahu a obsahu minimálního preventivního programu prevence rizikového chování pro základní školy]. Adiktologie.

[28] Miovsky M., Novak P., Stastna L., Gabrhelik R., Jurystova L., Vopravil J. (2012). Resultados del programa de prevención escolar Unplugged sobre el uso del tabaco en la República Checa. Adicciones.

[29] Faggiano F., Vigna-Taglianti F., Versino E., Zambon A., Borraccino A., Lemma P. (2005). School-based prevention for illicit drugs’ use. Cochrane Database Syst. Reviews..

[30] Gabrhelik R., Duncan A., Miovsky M., Furr-Holden C.D.M., Stastna L., Jurystova L. (2012). “Unplugged”: A school-based randomized control trial to prevent and reduce adolescent substance use in the Czech Republic. Drug Alcohol Depend..

[31] Gabrhelík R., Orosová O., Miovský M., Vonkova H., Berništerová M., Minařík J. (2014). Studying the effectiveness of school-based universal prevention interventions in the Czech Republic and Slovakia. Adiktologie.

[32] Miovský M., Voňková H., Gabrhelík R., Šťastná L. (2015). Universality Properties of School-Based Preventive Intervention Targeted at Cannabis Use. Prev. Sci..

[33] Gabrhelik R., Duncan A., Lee M.H., Stastna L., Furr-Holden C.D.M., Miovsky M. (2012). Sex specific trajectories in cigarette smoking behaviors among students participating in the Unplugged school-based randomized control trial for substance use prevention. Addict. Behav..

[34] Miovský M., Vonkova H., Čablová L., Gabrhelík R. (2015). Cannabis use in children with individualized risk profiles: Predicting the effect of universal prevention intervention. Addict. Behav..

[35] Vondráčková P., Gabrhelík R. (2016). Prevention of Internet addiction: A systematic review. J. Behav. Addict..

[36] Lakens D. (2013). Calculating and reporting effect sizes to facilitate cumulative science: A practical primer for t-tests and ANOVAs. Front. Psychol..

[37] Smahel D., Machackova H., Mascheroni G., Dedkova L., Staksrud E., Ólafsson K., Livingstone S., Hasebrink U. EU Kids Online 2020: Survey Results from 19 Countries. https://www.eukidsonline.ch/files/Eu-kids-online-2020-international-report.pdf.

[38] Rumpf H.J., Brand M., Wegmann E., Montag C.H., Müller A., Müller K., Wölfling K., Stark R., Steins-Löber S., Hayer T. (2020). The COVID-19 Pandemic and Behavioral Addiction-New Challenges for Structural and Behavioral Prevention. Sucht.

